# History-restricted marginal structural model and latent class growth analysis of treatment trajectories for a time-dependent outcome

**DOI:** 10.1515/ijb-2023-0116

**Published:** 2024-08-12

**Authors:** Awa Diop, Caroline Sirois, Jason R. Guertin, Mireille E. Schnitzer, James M. Brophy, Claudia Blais, Denis Talbot

**Affiliations:** Département de médecine sociale et préventive, Université Laval, Centre de recherche du CHU de Québec – Université Laval, Axe santé des populations et pratiques optimales en santé, Québec, QC, Canada; Faculté de pharmacie, Université Laval, Centre de recherche du CHU de Québec – Université Laval, Axe santé des populations et pratiques optimales en santé, Québec, QC, Canada; Tissue Engineering Laboratory (LOEX), Département de médecine sociale et préventive, Université Laval, Centre de recherche du CHU de Québec – Université Laval, Axe santé des populations et pratiques optimales en santé, Québec, QC, Canada; Faculté de pharmacie et Département de médecine sociale et préventive, ESPUM, Department of Epidemiology, Biostatistics, and Occupational Health, Université de Montréal, McGill University, Montréal, QC, Canada; Hospital Center for Health Outcomes Research, McGill University, Montréal, QC, Canada; Institut national de santé publique du Québec (INSPQ), Québec, QC, Canada

**Keywords:** pooled LTMLE, g-computation, IPTW, history-restricted MSMs, survival analysis, cardiovascular disease

## Abstract

In previous work, we introduced a framework that combines latent class growth analysis (LCGA) with marginal structural models (LCGA-MSM). LCGA-MSM first summarizes the numerous time-varying treatment patterns into a few trajectory groups and then allows for a population-level causal interpretation of the group differences. However, the LCGA-MSM framework is not suitable when the outcome is time-dependent. In this study, we propose combining a nonparametric history-restricted marginal structural model (HRMSM) with LCGA. HRMSMs can be seen as an application of standard MSMs on multiple time intervals. To the best of our knowledge, we also present the first application of HRMSMs with a time-to-event outcome. It was previously noted that HRMSMs could pose interpretation problems in survival analysis when either targeting a hazard ratio or a survival curve. We propose a causal parameter that bypasses these interpretation challenges. We consider three different estimators of the parameters: inverse probability of treatment weighting (IPTW), g-computation, and a pooled longitudinal targeted maximum likelihood estimator (pooled LTMLE). We conduct simulation studies to measure the performance of the proposed LCGA-HRMSM. For all scenarios, we obtain unbiased estimates when using either g-computation or pooled LTMLE. IPTW produced estimates with slightly larger bias in some scenarios. Overall, all approaches have good coverage of the 95 % confidence interval. We applied our approach to a population of older Quebecers composed of 57,211 statin initiators and found that a greater adherence to statins was associated with a lower combined risk of cardiovascular disease or all-cause mortality.

## 1 Introduction

Prevention of cardiovascular diseases (CVDs) is particularly important given their high prevalence and impact on population health [1]. However, to implement primary prevention strategies in the population, a rigorous measurement of expected benefits is necessary [2]. Statins, medications that reduce cholesterol levels, are used in the primary prevention of CVD events, but their benefit for this purpose among older individuals is uncertain [3, 4]. In this study, our goal is to measure the potential of actual patterns of time-varying statin usage to prevent a first CVD or death event using medical administrative databases. A widespread approach to measuring the impact of statins on CVD events is by estimating the effect of the cumulative number of periods that subjects were exposed to the treatment [5]. Alternatively, some researchers are more interested in knowing the effect of treatment adherence versus non adherence, i.e., compliance versus non compliance of the patient to the physician’s recommendations [6]. Adherence is often described using approaches such as the medication possession ratio or the proportion of days covered [7, 8]. However, these methods do not capture the complex dynamics of a time-varying treatment. Latent class growth analysis (LCGA) has been proposed as a better method to measure actual patterns of adherence [9].

In our previous work, we introduced a theoretical framework that combines LCGA and a nonparametric marginal structural model (LCGA-MSM) to measure the impact of groups of treatment trajectories (or trajectory groups) on an outcome measured at the end of the follow-up period [10]. The LCGA-MSM approach tackles some important challenges encountered when analysing longitudinal data. Indeed, the LCGA summarizes the numerous observed time-varying treatment patterns into a few trajectory groups [9–11]. The LCGA-MSM also deals adequately with time-varying variables that can have a double role as confounders and mediators when estimating the effect of these trajectory groups on the outcome. Such treatment-confounder feedback is expected in our application. For example, it is well known that diabetes is an important CVD risk factor [12, 13]. Moreover, in a meta-analysis, Sattar et al. [14] concluded that statin therapy is associated with a slightly increased risk of developing diabetes. Therefore, diabetes has a potential double role of confounder and mediator in the pathway between statins and CVDs. LCGA-MSM also gives a direct population-level causal interpretation of group effects on the outcome, an advantage of MSMs [15]. Thus, a combination of LCGA and MSM might contribute to better understanding the effects of treatment adherence and therefore help make better clinical or public health decisions. For example, LCGA-MSM may help determine whether benefits are expected among patients with imperfect adherence, and whether it is worth developing policies to improve adherence among such patients.

While the LCGA-MSM framework has multiple advantages, it has some limitations. One noteworthy limitation of the LCGA-MSM is the increasing time gap between the period during which treatment trajectories are evaluated and measurements of the outcome over the following period. Moreover, it may not currently be suitable for some complex applications in which the outcome is time-dependent. Indeed, our previous work focused on a treatment measured during a single window and an outcome measured only at the end of that window [10]. This may result in a loss of information since, in a real-life setting, events can occur during the course of the treatment trajectory. In this paper, we aim to generalize the LCGA-MSM framework that respects the time-dependent nature of the treatment and outcome. To do this, we propose combining a nonparametric history-restricted marginal structural model (HRMSM) with LCGA. HRMSMs are a generalization of standard MSMs that consist of a repeated application of standard MSMs on different time-windows [16–18].

Our work extends the current literature in several directions. First, we extend LCGA-MSMs to make possible the use of multiple measurements of the outcome through the HRMSM framework. To the best of our knowledge, we also present the first application of HRMSMs with a time-to-event outcome. It was previously noted that HRMSMs could pose important interpretation problems when targeting hazard ratios in a survival analysis context [16]. The causal parameter we propose circumvents this caveat. Finally, we introduce a pooled longitudinal targeted maximum likelihood estimation (LTMLE) estimator of the parameters of an HRMSM and an associated variance estimator based on the efficient influence curve that accounts for the correlations arising from the repeated use of the data. We consider three different estimators of the parameters of our proposed LCGA-HRMSM: inverse probability of treatment weighting (IPTW), g-computation, and the LTMLE. In the remainder, we first present the data used in our motivating illustration. We then present a review of LCGA-MSMs, the notation and data structure, the theoretical framework of LCGA-HRMSMs and the different estimators. Simulation studies are used to evaluate and compare the different estimators. We then return to the motivating real-data analysis before ending the paper with some recommendations for the practical application of our proposed approach.

## 2 Data

We built a retrospective cohort for the period between April 1, 2013 and March 30, 2018 using the Quebec integrated chronic disease surveillance system (QICDSS) available at the *Institut national de santé publique du Québec (INSPQ)*. The QICDSS is updated annually and is composed of five linked databases [19]: (1) health insurance registry, (2) hospitalization database, (3) vital statistics death (4) physician claims and (5) pharmaceutical services. All five databases are merged using the unique identifier of individuals that is their health insurance number [19]. We identified individuals aged greater than 65 years old on April 1, 2013 from Quebec, Canada. Because we are interested in a primary prevention context, only individuals without any CVD history in the last five years were included in the study. The quantification of CVD was based on an algorithm approved by the public health agency of Canada. Individuals with a statin claim in the year prior to enrolment were excluded as we are interested only in statin initiators. To be included, an individual was thus required to be enrolled in the public drug insurance plan for at least one year prior to enrolment and the following years. Thus, we included in the cohort 57,211 individuals who initiated statin treatment between April 2013 and September 2017. It is noteworthy that all Quebecers are enrolled in public drug insurance at the age of 65.

## 3 Review of LCGA-MSMs

In this section, we briefly present the LCGA-MSM approach (see Diop et al. [10] for more details). LCGA-MSM is a two-step approach. In the first step, an LCGA is used to classify individuals into *J* distinct trajectory groups *z*_1_, …, *z*_*J*_ based on their treatment trajectory [11]. LCGA is a mixture modeling approach that represents the treatment trajectory within each group as a polynomial or some other (e.g., cubic spline) function of time [11, 20], [21], [22]. In a second step, a working MSM is chosen to relate the outcome to these trajectory groups. Following [23], the causal parameter of interest *β* is defined as a projection of the true nonparametric MSM m* onto the working model *m*. From a practical point of view, LCGA first allows clustering the observed treatment trajectories in a few distinct groups. These groups allow a simplified visualization of the most commonly observed patterns of exposures over time. In a second step, the MSM allows estimating the effect of these trajectory groups as a best approximation to the true underlying causal model under the working model. The parameter of interest can be conceptualized as follows: “if we were to conduct a randomized trial with groups consisting of the possible individual treatment trajectories, and then cluster the treatment groups according to the trajectory groups found in the first step, how would the mean outcome differ between clusters?” [10].

Consider the data presented in Section 2 with follow-up times *t* = 1, …, *K* with *K* = 60 months, where *t* = 1 corresponds to the month of statin initiation (index date). An example of an LCGA-MSM application on these data would be to use data on time points *t* = 1, …, *K*′ (*K*′ < *K*) to classify individuals into trajectory groups and then use the remaining period (*t* = *K*′ + 1, …, *K*) as a follow-up for the outcome. For example, months 1–12 could be used to construct trajectories and months 13–60 for the outcome follow-up. For simplicity, we temporarily assume that events cannot occur during the exposure follow-up time, noting that our previous work [10] and the extension presented in later sections accommodate the occurrence of events during the exposure follow-up. In the following, we use capital letters to represent random variables and corresponding lower case letters to represent observed or fixed values these variables take. Let ${\bar{A}}_{t}=\left(A_{1},A_{2},\ldots ,A_{t}\right)$ be the treatment trajectory up to time *t* with $\bar{A}\equiv {\bar{A}}_{K^{\prime }}$ . We denote by *Y*_*K*_ the observed outcome that can be binary or continuous and by $Y_{K}^{\bar{a}}$ the counterfactual outcome under a specific treatment trajectory $\bar{a}$ , which is measured during the outcome follow-up time (between *K*′ + 1 and *K*). Similarly, ${\bar{L}}_{t}=\left(L_{1},L_{2},\ldots L_{t}\right)$ is the covariates’ history up to time *t* with $\bar{L}\equiv {\bar{L}}_{K^{\prime }}$ . We denote by *F*_*X*_ the unknown distribution of all possible counterfactual variables $X=\left({\bar{L}}^{\bar{a}},Y_{K}^{\bar{a}}:\bar{a}\in A\right)$ where A is the set of all possible values of $\bar{a}$ . We denote by $P_{F_{X}}$ the distribution of the observed data $O=\left\{Y_{K},{\bar{A}}_{K^{\prime }},{\bar{L}}_{K^{\prime }}\right\}$ , from which *i* = 1, …, *n* independent and identically distributed observations are randomly drawn. The following nonparametric form of an MSM (the true model) is considered:

(1) $$E_{F_{X}}\left(Y_{K}^{\bar{a}}\right)=m^{*}\left(\bar{a}\right).$$

The nonparametric identification of model (1) from the observed data can be achieved under the following causal assumptions: (1) no interference between subjects: the potential outcome of a given individual is not affected by others’ exposure; (2) positivity: for each level $\left({\bar{a}}_{t},{\bar{l}}_{t}\right)$ , $P\left(A_{t}=a_{t}|{\bar{A}}_{t-1}={\bar{a}}_{t-1},{\bar{L}}_{t}={\bar{l}}_{t}\right)>0$ . In other words, in each stratum defined by previous treatment and covariates, we find both exposed and unexposed individuals at time *t*. (3) sequential conditional exchangeability: there are no unmeasured confounders conditional on the history of treatment up to time *t* − 1 and the history of the covariates up to time *t* [15], that is $Y_{K}^{\bar{a}}⊥\;\;\;⊥A_{t}|{\bar{A}}_{t-1},{\bar{L}}_{t}$ ; (4) consistency: given the observed treatment trajectory, the observed outcome and the potential outcome under that given trajectory are the same, formally if $\bar{A}=\bar{a}$ then $Y_{K}^{\bar{a}}=Y_{K}$ . We note that although sequential conditional exchangeability is often interpreted as a “no unmeasured confounders” assumption, this assumption is also violated under collider stratification.

The parameter of interest is defined as a projection of the true nonparametric MSM m* onto a parametric working model that is a function of the trajectory groups. Let *z*_*i*_ be the trajectory group assigned to subject *i* as defined previously and $z^{*}\left(\bar{a}\right)=\left(z_{1}^{*}\left(\bar{a}\right),\ldots ,z_{J}^{*}\left(\bar{a}\right)\right)$ dummy variables indicating whether the individual trajectory $\bar{a}$ is clustered into trajectory group 1, …, *J*. For example, when the outcome *Y*_*K*_ is binary, the true model could be projected onto the following logistic LCGA-MSM:

(2) $$m\left(\bar{a}|\beta \right)\equiv E\left(Y_{K}^{\bar{a}}\right)=\text{expit}\left(\beta_{0}+\beta_{1}z_{1}^{*}\left(\bar{a}\right)+\beta_{2}z_{2}^{*}\left(\bar{a}\right)+\cdots +\beta_{J-1}z_{J-1}^{*}\left(\bar{a}\right)\right).$$

To define our parameter of interest *β* we also need to choose a loss function and a projection weight function $\lambda \left(\bar{a}\right)$ . For example, we consider the negative log-likelihood of a logistic regression. Denote by $M^{*}$ the infinite-dimensional space of all possible functions m* relating the counterfactual expectation with the treatment trajectories and $m\in M^{*}$ the working model. Considering our example working model and loss-function, the parameter is then defined as:

$$\beta \left(F_{X}|m,\lambda \right)=\underset{\beta \in R^{k}}{\mathrm{arg min}}\;E_{F_{X}}\left\{-\underset{\bar{a}\in \bar{A}}{\sum }\left[\log\left(m{\left(\bar{a}|\beta \right)}^{Y_{K}^{\bar{a}}}{\left(1-m\left(\bar{a}|\beta \right)\right)}^{\left(1-Y_{K}^{\bar{a}}\right)}\right)\right]\lambda \left(\bar{a}\right)\right\}.$$

In our previous work, we proposed an IPTW estimator of this parameter. The expression of the IPTW is given by:

(3) $$W\left(\bar{A}\right)=\frac{\lambda \left(\bar{A}\right)}{g\left(\bar{A}|X\right)},$$

where we define, $g\left(\bar{A}|X\right)=\prod_{t=1}^{K^{\prime }}P\left(A_{t}=1|{\bar{A}}_{t-1},{\bar{L}}_{t}\right)$ and $\lambda \left(\bar{A}\right)$ is a projection weight function. If stabilized weights are of interest, we set $\lambda \left(\bar{A}\right)=\prod_{t=1}^{K^{\prime }}P\left({\bar{A}}_{t}|{\bar{A}}_{t-1}\right)$ . For unstabilized weights $\lambda \left(\bar{A}\right)=1$ . Thus, the IPTW estimating function is given by:

(4) $$D_{h_{\lambda }}\left(O|\beta \right)=\frac{h_{\lambda }\left(\bar{A},L_{1}\right)\epsilon_{\bar{a}}\left(\beta \right)}{\overset{K^{\prime }}{\underset{t=1}{\prod }}P\left(A_{t}=1|{\bar{A}}_{t-1},{\bar{L}}_{t}\right)},$$

with $h_{\lambda }\left(\bar{A}\right)\equiv \lambda \left(\bar{A}\right)\frac{\partial }{\partial \beta }m\left(\bar{a}|\beta \right)\times m\left(\bar{a}|\beta \right)$ , $\lambda \left(\bar{A}\right)=\prod_{t=0}^{K^{\prime }}P\left({\bar{A}}_{t}|{\bar{A}}_{t-1}\right)$ and $\epsilon_{\bar{a}}\left(\beta \right)=Y_{K}^{\bar{a}}-m\left(\bar{a}|\beta \right)$ .

In practice, parameters of the working model can be directly estimated using a weighted logistic regression of *Y*_*K*_ on *z* with observations weighted according to the weights defined in Equation (3). The logistic regression model can be fitted using a weighted *generalized estimating equations* (GEE) estimator, with, for example, the function *geeglm* from the **R** package *geepack*. A simple practical solution for inference is to use the sandwich variance estimator of the GEE routine, which treats the weights as known and results in conservative statistical inferences [24]. Alternative solutions for inferences include (a) bootstrapping the estimation of the weights and the GEE regression, but not the LCGA, or (b) stacking the estimating equations of the weights and the GEE regression [see e.g., 25], both solutions providing consistent estimation of the variance. Note that the distribution of the trajectory groups is only a function of the joint distribution of the treatments $\bar{A}$ and does not depend on the parameter of interest *β*; thus *z* is an ancillary statistic [10, 26, 27]. Therefore, the data-driven estimation of the trajectory groups can be ignored when estimating the parameters of our LCGA-MSM. However, it should be noted that inferences are conditional on the selected LCGA. As argued by multiple authors, clustering that does not involve using the outcome does not invalidate inferences [28, 29]. Methods that assess the sensitivity of the results to the uncertainty of subjects’ classification to trajectory groups have been developed and could be considered within the LCGA-MSM framework [30].

**Figure 1:**
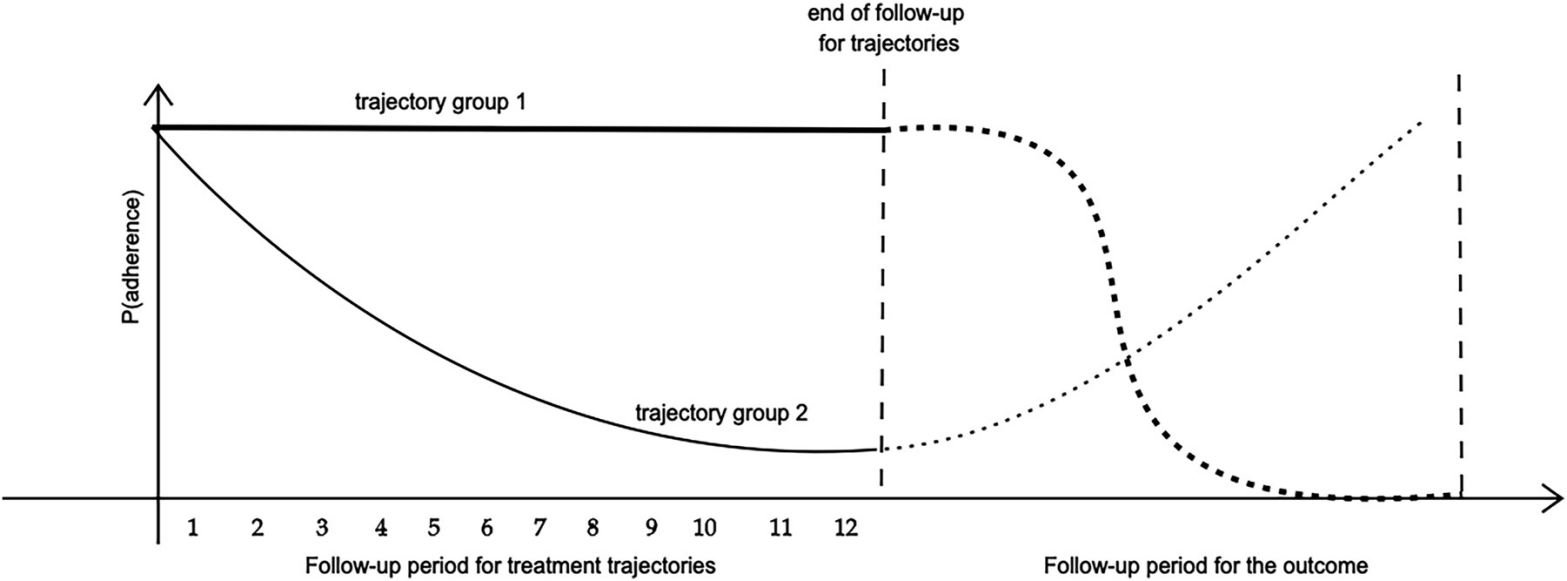
Illustration of LCGA-MSM after the follow-up period of treatment trajectories.

In Figure 1, we present an example in which adherence changes considerably during the follow-up period of the outcome. In an LCGA-MSM analysis, the outcome at any point of the follow-up would be modeled as a function of the trajectory determined using the single treatment window. Opposingly, the LCGA-HRMSM framework we introduce next considers multiple treatment windows and allows the subjects’ trajectory groups to change from one window to another. Consequently, the outcome at any time point can be modeled as a function of the treatment trajectory group in the most recent treatment window.

**Figure 2:**
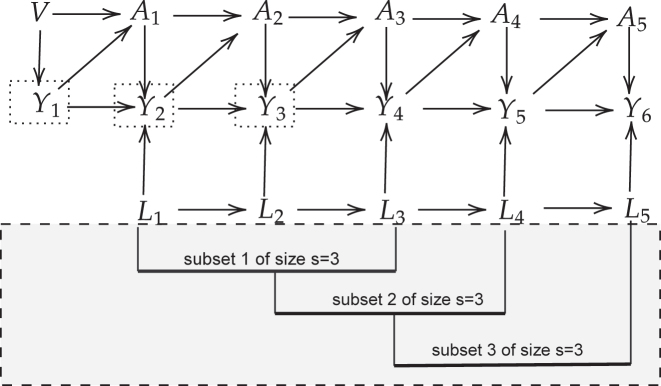
Simplified causal graph for a follow-up of length *K* = 6 and for subsets of length *s* = 3. Associations between the time-varying covariates (*L*_1_, *L*_2_, *L*_3_, *L*_4_ and *L*_5_) and the time-varying treatment (*A*_1_, *A*_2_, *A*_3_, *A*_4_ and *A*_5_) were omitted to avoid overloading the figure. Similarly, unmeasured common causes between *L* and *Y* nodes or arrows from *Y* nodes to *L* nodes are allowed but omitted. Note that *V* represents baseline characteristics.

In the example in Figure 2, if we were to apply an LCGA-MSM, we would consider that the outcome is observed at the end of the follow-up at *K* = 6 which might not be the best use of the data. We also see in Figure 2 that events might be observed during the exposure measurement period. That is, the treatment trajectory does not necessarily precede the outcome; instead they are two concomitant phenomena. HRMSMs can thus be useful to make the most of the available information.

## 4 History restricted MSM and LCGA

HRMSMs can be seen as a repeated application of a standard MSM to different subsets of the original data across time points. More precisely, estimating the parameters of an HRMSM first requires splitting the data into multiple windows of fixed size. For each window, the counterfactual outcome at the end of the window is modeled as a function of the treatments measured during that window. This process is performed simultaneously for all windows, thus resulting in repeated measurements of both the trajectory groups and the outcome. We emphasize that our focus is on HRMSMs for which there is a single baseline for each outcome time point. HRMSMs with multiple baselines for each outcome time-point are susceptible to theoretical challenges [31]. In the following, we present the notation in the t-specific framework used for HRMSMs in [16], the data structure, the LCGA in the t-specific framework and, the definition and identification of the causal parameter of interest before giving a definition of HRMSMs.

### 4.1 Notation in the t-specific framework

In the conventional counterfactual framework, the data are considered from baseline to the end of the follow-up as one set. In HRMSMs, a new form of counterfactual framework is defined: the t-specific (for time-specific) counterfactual framework [16]. In this t-specific framework, multiple time intervals of fixed history size *s* are chosen from the original data according to the context of the study. We define $T_{s}$ , the set indexing all time intervals. When the total follow-up is *K*, we can form *K* − *s* + 1 time intervals of length *s*. For example, if *K* = 60 and *s* = 6 months as in our application, we can form 55 overlapping time intervals: [1, 6], [2, 7], [3, 8] … [53, 58], [54, 59], [55, 60]; therefore, $T_{s}=\left\{1,2,\ldots ,55\right\}$ .

We denote by ${\bar{A}}_{d}\equiv {\bar{A}}_{d,d+s-1}=\left\{A_{d},\ldots ,A_{d+s-1}\right\}$ the treatment trajectories in the *d*th time interval *I*_*d*_ = [*d*, *d* + *s* − 1]. Similarly, ${\bar{L}}_{d,d+s-1}$ denote the covariates’ history in the *d*th interval of follow-up, i.e., the history up to the time point *d* + *s* − 1. We denote by $Y_{d+s}^{{\bar{a}}_{d}}$ the counterfactual outcome measured at the end of the time interval *I*_*d*_ and corresponding to a treatment trajectory ${\bar{a}}_{d}$ between time points *d* and *d* + *s* − 1. In the following, our outcome is the occurrence of a first event in line with our application regarding primary prevention but our approach can easily be extended to a setting with repeated events. In our application, the outcome $Y_{d+s}^{{\bar{a}}_{d}}=1$ if an event would have counterfactually occurred under the treatment regime ${\bar{a}}_{d}$ and 0 otherwise. Similarly, ${\bar{L}}^{{\bar{a}}_{d}}\equiv {\bar{L}}_{d,d+s-1}^{{\bar{a}}_{d}}$ denotes a counterfactual covariate process in the *d*th interval. We denote by *F*_*X*_ the unknown distribution of all possible counterfactual variables $X=\left({\bar{L}}^{{\bar{a}}_{d}},Y_{d+s}^{{\bar{a}}_{d}}:{\bar{a}}_{d}\in A_{d}\right)$ where $A_{d}$ is the set of all possible values of ${\bar{a}}_{d}$ for the *d*th interval. The exposure at each time *t* in the *d*th time interval is considered as bivariate *A*_*d*,*t*_ = (*A*_*d*,*t*_(1), *A*_*d*,*t*_(2)). The first exposure *A*_*d*,*t*_(1) represents the statin intake. Thus *A*_*d*,*t*_(1) = 1 indicates that the subject did take their prescriptions at time *t* in the *d*th time interval and *A*_*d*,*t*_(1) = 0 otherwise. The second exposure, *A*_*d*,*t*_(2) is a censoring variable and takes the value 1 if there is right censoring (e.g., end of follow-up or loss to follow-up). When *A*_*d*,*t*_(2) = 1, all subsequent variables of the subject are treated as missing. If *A*_*d*,*t*_(2) = 0, the subject is uncensored in the *d*th time interval. As will be seen shortly, *A*_*d*,*t*_(2) will be used to account for individuals with incomplete exposure follow-up time, either because of loss to follow-up or because an outcome occurred during the exposure follow-up time. This will allow avoiding the potential selection bias that may arise because trajectory groups can only be determined for people with complete exposure follow-up.

### 4.2 Data structure of HRMSMs

In the following, we present the organization of the data in the context of HRMSMs. For simplicity, we consider a data-generating mechanism adapted from Schnitzer et al. [32]. The follow-up time is of length *K* = 6 and the time-dependent outcome is observed at each time *t* = 1, 2, …, 6 (see Figure 2). However, only outcomes *Y*_4_, *Y*_5_ and *Y*_6_, measured at the end of each subset of length *s* = 3, are considered as dependent variables; indeed a period of three measures has to be spared to construct treatment trajectories [33].

In a typical longitudinal causal inference problem, the observed data are usually represented as: $O=\left\{Y_{1},L_{1},A_{1},Y_{2},L_{2},A_{2},Y_{3},L_{3},A_{3},Y_{4},L_{4},A_{4},Y_{5},L_{5},A_{5},Y_{6}\right\}$ where *L*_1_, *L*_2_, *L*_3_, *L*_4_, *L*_5_ represent the time-varying covariates, *A*_1_, *A*_2_, *A*_3_, *A*_4_, *A*_5_ the bivariate treatment indicators and *Y*_1_, *Y*_2_, *Y*_3_, *Y*_4_, *Y*_5_, *Y*_6_ the time-dependent outcome. This representation of the data entails temporal ordering. The baseline outcome is *Y*_1_ = 0 for all individuals. Put differently, all individuals were at risk of experiencing the event at the beginning of the follow-up.

**Figure 3:**
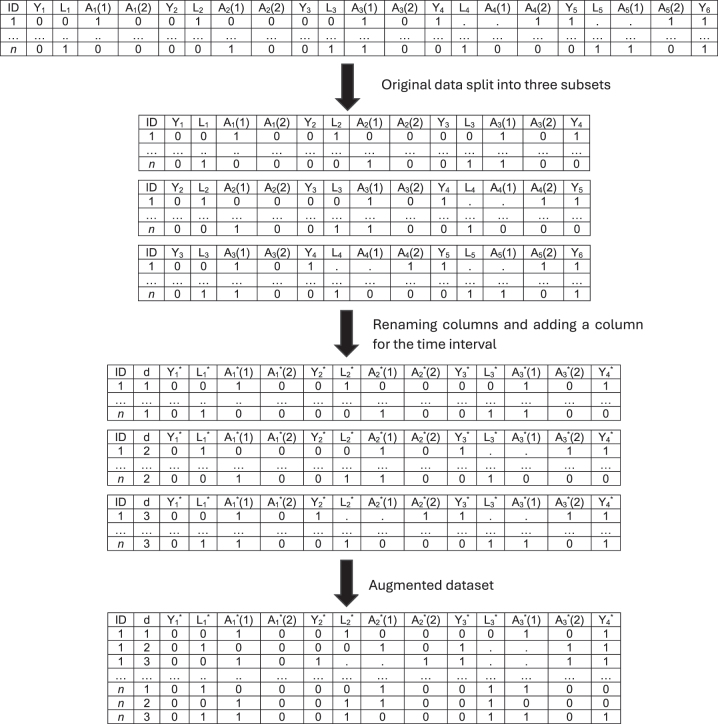
Example of data structure of HRMSMs for a follow-up of length *K* = 5 and with binary variables.

In an HRMSM, the data are rearranged in an augmented dataset that includes a separate row for each interval to which a subject contributes. Continuing the previous example, we get three subsets from the original data: *O* ^1^ = {*Y*_1_, *L*_1_, *A*_1_, *Y*_2_, *L*_2_, *A*_2_, *Y*_3_, *L*_3_, *A*_3_, *Y*_4_}, *O* ^2^ = {*Y*_2_, *L*_2_, *A*_2_, *Y*_3_, *L*_3_, *A*_3_, *Y*_4_, *L*_4_, *A*_4_, *Y*_5_} and *O* ^3^ = {*Y*_3_, *L*_3_, *A*_3_, *Y*_4_, *L*_4_, *A*_4_, *Y*_5_, *L*_5_, *A*_5_, *Y*_6_}. In a general form, the observed data are represented as *K* − *s* + 1 data structures *O* ^*d*^: $O^{d}=\left\{{\bar{A}}_{d,d+s-1},{\bar{L}}_{d,d+s-1},{\bar{Y}}_{d,d+s}\right\}$ with distribution $P_{F_{X}}^{d},d=1,\ldots ,K-s+1$ . In the t-specific counterfactual framework, these subsets are considered simultaneously [16]. Denote by $L_{t}^{*}$ , $A_{t}^{*}$ , the covariates, the exposure and the outcome at the *t*th time point of a given interval, after rearranging the data. The following data structure represents the augmented version of the data: $O=\left\{Y_{1}^{*},=,\left(Y_{1},Y_{2},Y_{3}\right),,,L_{1}^{*},=,\left(L_{1},L_{2},L_{3}\right),,,A_{1}^{*},=,\left(A_{1},A_{2},A_{3}\right),,,Y_{2}^{*},=,\left(Y_{2},Y_{3},Y_{4}\right),,,L_{2}^{*},=,\left(L_{2},L_{3},L_{4}\right),,,A_{2}^{*},=,\left(A_{2},A_{3},A_{4}\right),,,Y_{3}^{*}\right.\left.=,\left(Y_{3},Y_{4},Y_{5}\right),,,L_{3}^{*},=,\left(L_{3},L_{4},L_{5}\right),,,A_{3}^{*},=,\left(A_{3},A_{4},A_{5}\right),,,Y_{4}^{*},=,\left(Y_{4},Y_{5},Y_{6}\right)\right\}$ . Figure 3 summarizes this process. Note that because we require a complete exposure follow-up, individuals with events occurring during the exposure follow-up are considered as censored, that is, if $Y_{t}^{*}=1$ then $A_{t}^{*}\left(2\right)=1$ . Censoring could also occur in absence of an event during the exposure follow-up if there is a loss to follow-up. Also note that in the following, we only present quantities using the “original” wide format data notation, not the transformed augmented data notation.

### 4.3 LCGA in the t-specific framework

After rearranging the data in an augmented dataset, the observed treatment trajectories measured in all time intervals are simultaneously summarized into *J* distinct trajectory groups *z*_1_, …, *z*_*J*_ using LCGA [11]. In other words, an LCGA is applied to the entire pooled data *O* ^*d*^, *d* = 1, …, *K* − *s* + 1, simultaneously. This procedure leads to a repeated version of the LCGA. Note that individuals can get assigned to different trajectory groups in different subsets. In the setting where individuals can be lost to follow-up, the LCGA is applied to the available data and the missing data mechanism is considered to be missing at random (MAR) [34]. Note that medico-administrative databases cover the vast majority of the population of interest and there are thus very few losses to follow-up.

### 4.4 Definition of nonparametric HRMSMs

**Figure 4:**
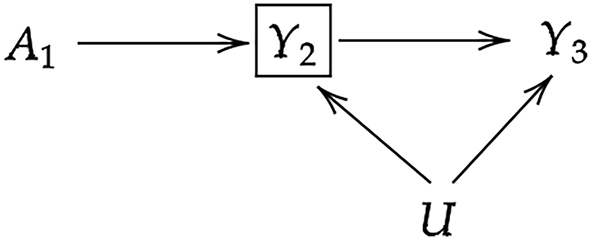
Illustration of the built-in bias problem of HRs with two time points.

Given that we are interested in a time-to-event outcome, it may seem natural to define our nonparametric HRMSM as a model for the hazard ratio (HR). However, HRs lead to two problems of interpretation as discussed by Neugebauer et al. [16]. The first problem is that HRs have a well known built-in selection bias [35]. In our context, one possible scenario where selection bias can occur with HRs, is when there exists an unmeasured risk factor *U* of cardiovascular diseases (*Y*) as illustrated through the directed acyclic graph (DAG) in Figure 4. Estimation of the HR at the second period of follow-up is conditional on being event-free at the first period of follow-up (*Y*_2_ = 0). This conditioning is expected to result in an underestimation of the benefits of a preventive treatment, like statins, at the second period of follow-up. This selection bias is induced by what Hernán [35] called differential selection of less susceptible individuals to the risk factor *U* or differential depletion of susceptible individuals to the risk factor *U*. Indeed, individuals most at risk are going to experience the event quicker in the control group than in the treatment group, since the treatment helps to compensate for the increased risk of these individuals. Thus, at the second period, the treatment group differs from the control group with respect to the risk factor *U*: the treatment group comprises a greater proportion of individuals at high risk than the control group.

Because of the limitations of HRs as an effect measure, the survival curve or its complement, the cumulative risk, have been recommended [35]. In a standard MSM, survival curves under different trajectories can be constructed using HRs, allowing the comparison of survival curves between treatment trajectories. This leads us to the second problem. Indeed in HRMSMs, it is impossible to construct a survival curve using HRs [16]. In fact, because the trajectory group may change from one window to another, the HR at a given time point *t* does not correspond to a single trajectory group. To bypass these problems, we propose to use a model for the absolute risk. More precisely, we propose the following form for the nonparametric HRMSM with a time-dependent outcome for the *d*th interval *I*_*d*_ = [*d*, *d* + *s* − 1]:

(5) $$E_{F_{X}}\left[Y_{d+s}^{{\bar{a}}_{d}}|Y_{d}=0\right]=m^{*}\left(Y_{d}=0\right).$$

For the first interval, the absolute risk corresponds to the number of individuals that experienced the event in the interval *I*_1_ divided by the number of individuals at risk at the beginning of the interval *I*_1_ under the counterfactual regime of treatment ${\bar{A}}_{1,s}\left(1\right)={\bar{a}}_{1,s}\left(1\right)$ with a counterfactual intervention ${\bar{A}}_{1,s}\left(2\right)=0$ that prevents censoring or events from happening during the exposure follow-up period for all individuals. The nonparametric HRMSM is thus defined as $P\left(Y_{s+1}^{{\bar{a}}_{1,s}\left(1\right),{\bar{a}}_{1,s}\left(2\right)=0}=1|Y_{1}=0\right)\equiv P\left(Y_{s+1}^{{\bar{a}}_{1,s}}=1\right)$ as ${\bar{a}}_{1,s}\equiv \left({\bar{a}}_{1,s}\left(1\right),{\bar{a}}_{1,s}\left(2\right)\right)$ . In the second interval *I*_2_ = [2, *s* + 1], treatment at time point *t* = 1 (*a*_1_) is left random (it is not part of the intervention defined by ${\bar{a}}_{2,s+1}$ ). Therefore, we have to condition on *Y*_2_ = 0 to ensure that we still have a population at risk at the beginning of the interval *I*_2_. The nonparametric HRMSM is $P\left(Y_{s+2}^{{\bar{a}}_{2,s+1}\left(1\right),{\bar{a}}_{2,s+1}\left(2\right)=0}=1|Y_{2}=0\right)\equiv P\left(Y_{s+2}^{{\bar{a}}_{2,s+1}}=1|Y_{2}=0\right)$ . The risk in the interval *I*_2_ is measured as the number of individuals that experienced the event divided by the number of individuals still at risk at the beginning of interval *I*_2_ under the treatment regime ${\bar{A}}_{2,s+1}\left(1\right)={\bar{a}}_{2,s+1}\left(1\right)$ and where ${\bar{A}}_{2,s+1}\left(2\right)=0$ prevents the occurrence of censoring or events during the follow-up period *I*_2_. For the *d*th interval *I*_*d*_ = [*d*, *d* + *s* − 1], the nonparametric HRMSM is $P\left(Y_{s+d}^{{\bar{a}}_{d,d+s-1}\left(1\right),{\bar{a}}_{d,d+s-1}\left(2\right)=0}=1|Y_{d}=0\right)\equiv P\left(Y_{s+d}^{{\bar{a}}_{d,d+s-1}}=1|Y_{d}=0\right)$ .

Assumptions in the t-specific counterfactual framework are similar to those described for the usual counterfactual framework. The main difference is that each assumption is made relative to each interval or t-specific set of data (see Appendix Section 10.1). Under these assumptions, the nonparametric HRMSM defined in Equation (5) is identified from the observed data as:

(6) $$\begin{array}{ll} & E_{F_{X}}\left(Y_{d+s}^{{\bar{a}}_{d}}|Y_{d}=0\right)=\underset{l_{d}}{\int }\ldots \underset{l_{d+s-1}}{\int }E\left(Y_{d+s}|Y_{d}=0,{\bar{A}}_{d,d+s-1}\left(1\right)={\bar{a}}_{d,d+s-1}\left(1\right),{\bar{A}}_{d,d+s-1}\left(2\right)=0,{\bar{L}}_{d,d+s-1}\right)\\ & \;\;\;\;\;\;\;f\left(l_{d+s-1}|{\bar{l}}_{d,d+s-2},{\bar{a}}_{d,d+s-2}\right)d\mu \left(L_{d+s-2}\right),\ldots f\left(l_{d}\right)d\mu \left(L_{d}\right), \end{array}$$

where *μ*s are dominating measures. HRMSMs are a class of MSMs with multiple advantages like a gain of statistical power. In addition, HRMSM allows investigating effect modification according to time-dependent baseline covariates since multiple baselines are considered simultaneously [16].

## 5 Definition and estimation of the parameters of the LCGA-HRMSM

### 5.1 Definition

Our causal parameter of interest *β* is defined as the vector that minimizes the statistical distance between the true causal marginal risk and the marginal risk defined through a working model, using a negative log-likelihood loss function. The statistical estimation problem is defined as the projection of the true model (the nonparametric HRMSM) $m^{*}\left[Y_{d}=0\right]\equiv \mu_{d}^{{\bar{a}}_{d}}$ onto the working model $m\left({\bar{a}}_{d}|\beta \right)$ with projection weights $\lambda \left({\bar{a}}_{d}\right)$ under all values of ${\bar{a}}_{d}$ . Because the nonparametric HRMSM is a model for the absolute risk, a natural choice for the working model is a log-linear model, such that the exponential of its coefficients can be interpreted as relative risks. For such a log-linear model, two common choices to define the loss function are the log-binomial or Poisson negative log-likelihoods. One potential challenge with the log-binomial likelihood is the issue where the common maximum likelihood algorithms failing to converge because predicted probabilities must remain between 0 and 1, which is not ensured by the log-link [36]. The Poisson log-likelihood does not share this limitation, but may yield predicted probabilities greater than 1 [36, 37]. Both loss functions are presented in the following. A specific example of such a log-linear working model is:

(7) $$\log\left(m\left({\bar{a}}_{d}|\beta \right)\right)=\beta_{0,d}+\beta_{1}z_{1}^{*}\left({\bar{a}}_{d}\right)+\beta_{2}z_{2}^{*}\left({\bar{a}}_{d}\right)+\cdots +\beta_{J-1}z_{J-1}^{*}\left({\bar{a}}_{d}\right).$$

Denoting $z^{*}\left({\bar{a}}_{d}\right)=\left[1,z_{1}^{*}\left({\bar{a}}_{d}\right),z_{2}^{*}\left({\bar{a}}_{d}\right),\ldots ,z_{J-1}^{*}\left({\bar{a}}_{d}\right)\right]$ , the working model can thus be rewritten:

(8) $$\log\left(m\left({\bar{a}}_{d}|\beta \right)\right)=z^{*}\left({\bar{a}}_{d}\right)\beta .$$

Formally, the causal parameter of interest is defined as:

$$\beta_{m,\lambda }=\underset{\beta }{\mathrm{arg min}}\;\;E\left[L_{\lambda }\left(\mu_{d}^{{\bar{a}}_{d}},m\left({\bar{a}}_{d}|\beta \right)\right)\right],$$

where $L_{\lambda }$ is a loss function that depends on projection weights $\lambda \left({\bar{a}}_{d}\right)$ . We consider next the full data estimating equations that arise from considering either the log-binomial or the Poisson negative log-likelihood as loss-functions. Intuitively, the estimates of *β* are chosen such that the working model is as close as possible to the true model. The target parameter of interest pools parameters beta over all time intervals, putting an equal weight on each.

#### 5.1.1 Log-binomial negative log-likelihood loss

The log-binomial likelihood and log-likelihood are respectively given by:

$$L\left(\beta |\bar{a}\right)\propto \underset{d\in T_{s}}{\prod }\overset{n}{\underset{i=1}{\prod }}m{\left({\bar{a}}_{d}|\beta \right)}^{y_{i,d+s}^{{\bar{a}}_{d}}}{\left(1-m\left({\bar{a}}_{d}|\beta \right)\right)}^{1-y_{i,d+s}^{{\bar{a}}_{d}}},$$

$$\begin{array}{c} \log\left(L\left(\beta |\bar{a}\right)\right)\propto \underset{d\in T_{s}}{\sum }\overset{n}{\underset{i=1}{\sum }}y_{i,d+s}^{{\bar{a}}_{d}}\log\left(m({\bar{a}}_{d}|\beta \right)+\left(1-y_{i,d+s}^{{\bar{a}}_{d}}\right)\log\left(1-m\left({\bar{a}}_{d}|\beta \right)\right) \end{array}.$$

The parameter of interest is defined as:

(9) $$\beta =\underset{\beta \in R^{k}}{\mathrm{arg min}}\;E_{F_{X}}\left\{-\underset{d\in T_{s}}{\sum }\underset{{\bar{a}}_{d}\in \bar{A}}{\sum }\left[Y_{d+s}^{{\bar{a}}_{d}}\log(m\left({\bar{a}}_{d}|\beta \right)+\left(1-Y_{d+s}^{{\bar{a}}_{d}}\right)\log\left(1-m\left({\bar{a}}_{d}|\beta \right)\right)\right]\lambda \left({\bar{a}}_{d}\right)\right\}.$$

The score *D*(*X*|*β*) is given by a first order derivative of the loss function defined in Equation (9). Therefore the score is given by:

(10) $$D\left(X|\beta \right)=E_{F_{X}}\left\{-\underset{d\in T_{s}}{\sum }\underset{{\bar{a}}_{d}\in {\bar{A}}_{d}}{\sum }\frac{\partial }{\partial \beta }m\left({\bar{a}}_{d}|\beta \right)\left[\frac{Y_{d+s}^{{\bar{a}}_{d}}-m\left({\bar{a}}_{d}|\beta \right)}{m\left({\bar{a}}_{d}|\beta \right)\left(1-m\left({\bar{a}}_{d}|\beta \right)\right)}\right]\lambda \left({\bar{a}}_{d}\right)\right\}.$$

Inserting $m\left({\bar{a}}_{d}|\beta \right)=e^{z^{*}\left({\bar{a}}_{d}\right)\beta }$ in Equations (9) and (10), we get:

(11) $$\beta =\underset{\beta \in R^{k}}{\mathrm{arg min}}\;E_{F_{X}}\left\{-\underset{d\in T_{s}}{\sum }\underset{{\bar{a}}_{d}\in \bar{A}}{\sum }\left[Y_{d+s}^{{\bar{a}}_{d}}\log\left(e^{z^{*}\left({\bar{a}}_{d}\right)\beta }\right)+\left(1-Y_{d+s}^{{\bar{a}}_{d}}\right)\log\left(1-e^{z^{*}\left({\bar{a}}_{d}\right)\beta }\right)\right]\lambda \left({\bar{a}}_{d}\right)\right\},$$

(12) $$D\left(X|\beta \right)=E_{F_{X}}\left\{-\underset{d\in T_{s}}{\sum }\underset{{\bar{a}}_{d}\in {\bar{A}}_{d}}{\sum }z^{*}\left({\bar{a}}_{d}\right)\left[\frac{Y_{d+s}^{{\bar{a}}_{d}}-e^{z^{*}\left({\bar{a}}_{d}\right)\beta }}{e^{z^{*}\left({\bar{a}}_{d}\right)\beta }\left(1-e^{z^{*}\left({\bar{a}}_{d}\right)\beta }\right)}\right]\lambda \left({\bar{a}}_{d}\right)\right\}.$$

#### 5.1.2 Poisson negative log-likelihood loss

The Poisson likelihood and log-likelihood are:

$$L\left(\beta |\bar{a}\right)=\underset{d\in T_{s}}{\prod }\overset{n}{\underset{i=1}{\prod }}\frac{e^{y_{i}^{\bar{a}}\log\left(m\left({\bar{a}}_{d}|\beta \right)\right)}e^{-m\left({\bar{a}}_{d}|\beta \right)}}{y_{i,d+s}^{{\bar{a}}_{d}}!}$$

$$\log\left(L\left(\beta |\bar{a}\right)\right)=\underset{d\in T_{s}}{\sum }\overset{n}{\underset{i=1}{\sum }}y_{i,d+s}^{{\bar{a}}_{d}}\log\left(m\left({\bar{a}}_{d}|\beta \right)\right)-m\left({\bar{a}}_{d}|\beta \right)-\log\left(y_{i,d+s}^{{\bar{a}}_{d}}!\right).$$

The parameter of interest is defined as:

(13) $$\beta =\underset{\beta \in R^{k}}{\mathrm{arg min}}\;E_{F_{X}}\left\{-\underset{d\in T_{s}}{\sum }\underset{{\bar{a}}_{d}\in \bar{A}}{\sum }\left[Y_{d+s}^{{\bar{a}}_{d}}\log\left(m\left({\bar{a}}_{d}|\beta \right)\right)-m\left({\bar{a}}_{d}|\beta \right)\right]\lambda \left({\bar{a}}_{d}\right)\right\}.$$

The score equation *D*(*X*|*β*) is given by a first order derivative of the loss function defined in Equation (13). Thus, we have:

(14) $$D\left(X|\beta \right)=E_{F_{X}}\left\{-\underset{d\in T_{s}}{\sum }\underset{{\bar{a}}_{d}\in {\bar{A}}_{d}}{\sum }\frac{\partial }{\partial \beta }\left[Y_{d+s}^{{\bar{a}}_{d}}\log\left(m\left({\bar{a}}_{d}|\beta \right)\right)-m\left({\bar{a}}_{d}|\beta \right)\right]\lambda \left({\bar{a}}_{d}\right)\right\}.$$

Inserting $m\left({\bar{a}}_{d}|\beta \right)=e^{z^{*}\left({\bar{a}}_{d}\right)\beta }$ in Equation (13) and $\frac{\partial }{\partial \beta }m\left({\bar{a}}_{d}|\beta \right)=z^{*}e^{z^{*}\left({\bar{a}}_{d}\right)\beta }$ in Equation (14), we get the expression of the loss function and the score equation:

(15) $$\beta =\underset{\beta \in R^{k}}{\mathrm{arg min}}\;E_{F_{X}}\left\{-\underset{d\in T_{s}}{\sum }\underset{{\bar{a}}_{d}\in \bar{A}}{\sum }\left[Y_{d+s}^{{\bar{a}}_{d}}z^{*}\left({\bar{a}}_{d}\right)\beta -e^{z^{*}\left({\bar{a}}_{d}\right)\beta }\right]\lambda \left({\bar{a}}_{d}\right)\right\}$$

⇔

(16) $$D\left(X|\beta \right)=E_{F_{X}}\left\{-\underset{d\in T_{s}}{\sum }\;\underset{{\bar{a}}_{d}\in {\bar{A}}_{d}}{\sum }z^{*}\left({\bar{a}}_{d}\right)\left[Y_{d+s}^{{\bar{a}}_{d}}-e^{z^{*}\left({\bar{a}}_{d}\right)\beta }\right]\lambda \left({\bar{a}}_{d}\right)\right\}.$$

### 5.2 Estimation

In what follows, we present three different estimators of the parameters of the LCGA-HRMSM: IPTW, g-computation and pooled LTMLE. Inferences for each estimator take into account that the same individual can contribute multiple observations in the augmented dataset.

#### 5.2.1 IPTW estimator

Based on the work of Neugebauer and van der Laan [23] and Neugebauer et al. [16], we first propose an IPTW estimator of the parameters of our LCGA-HRMSM. Under the sequential conditional exchangeanbility assumption, the expression of the IPTW estimator for the *d*th interval is equivalent to the expression of the IPTW estimator in the LCGA-MSM and is given by:

$$D_{h_{\lambda_{d}}}\left(O|\beta \right)=\frac{h_{\lambda_{d}}\left({\bar{a}}_{d}\right)\epsilon_{{\bar{a}}_{d}}\left(\beta \right)}{\overset{d+s-1}{\underset{j=d}{\prod }}g_{d}\left(A_{d,j}\left(1\right)\right)g_{d}\left(A_{d,j}\left(2\right)\right)},$$

where for the *d*th interval, $g_{d}\left(A_{d,j}\left(1\right)\right)g_{d}\left(A_{d,j}\left(2\right)\right)=P\left(A_{d,j}\left(1\right)|{\bar{A}}_{d,j-1}\left(1\right),A_{d,j}\left(2\right)=0,{\bar{L}}_{d,j},Y_{d}=0\right)P\left(A_{d,j}\left(2\right)=0|{\bar{A}}_{d,j-1}\left(1\right),{\bar{A}}_{d,j-1}\left(2\right)=0,{\bar{L}}_{d,j},Y_{d}=0\right)$ , $h_{\lambda_{d}}\left({\bar{a}}_{d}\right)\equiv \lambda_{d}\left({\bar{a}}_{d}\right)\frac{\partial }{\partial \beta }m\left({\bar{a}}_{d}|\beta \right)\times m\left({\bar{a}}_{d}|\beta \right)$ and $\epsilon_{{\bar{a}}_{d}}\left(\beta \right)=Y_{d+s}^{{\bar{a}}_{d}}-m\left({\bar{a}}_{d}|\beta \right)$ . The IPTW estimator pooled over time intervals is given by: $D_{h_{\lambda }}\left(O|\beta \right)=\sum_{d\in T_{s}}\frac{h_{\lambda_{d}}\left({\bar{a}}_{d}\right)\epsilon_{{\bar{a}}_{d}}\left(\beta \right)}{\prod_{j=d}^{d+s-1}g_{d}\left(A_{d,j}\left(1\right)\right)g_{d}\left(A_{d,j}\left(2\right)\right)}$ . In the case of a log-binomial model, $m\left({\bar{a}}_{d}|\beta \right)={\left[m\left({\bar{a}}_{d}|\beta \right)\left(1-m\left({\bar{a}}_{d}|\beta \right)\right)\right]}^{-1}$ and in the case of a Poisson model ${\mathrm{Var}}^{-1}m\left({\bar{a}}_{d}|\beta \right)={\left[m\left({\bar{a}}_{d}|\beta \right)\right]}^{-1}$ . The IPTW estimating equations are: $\sum_{d\in T_{s}}\sum_{i=1}^{n}D_{h_{\lambda_{d}}}\left(O_{i}|g_{d,n},\lambda_{d,n},\beta \right)=0$ . Under regularity conditions, the IPTW estimator of *β* is consistent and asymptotically linear if *λ*_*d*,*n*_ and *g*_*d*,*n*_ are consistent estimators of *λ*_*d*_ and *g*_*d*_ [38]. In other words, the causal risk can be estimated consistently using the IPTW estimator if the model for the treatment and the model for the censoring are correctly specified and we have a consistent estimator of the projection weight *λ*_*d*_.

**Table:** 

Fitting Procedure: LCGA-HRMSM with IPTW
In practice, implementation of the IPTW estimator follows these steps:
**Step 1:** Under the sequential conditional exchangeability assumption, estimate the treatment probability for the *d*th subset:
$\begin{array}{ll} g_{d}\left(A_{d,j}\left(1\right)\right)g_{d}\left(A_{d,j}\left(2\right)\right) & =\overset{d+s-1}{\underset{j=d}{\prod }}P\left(A_{d,j}\left(1\right)|{\bar{A}}_{d,j-1}\left(1\right),A_{d,j}\left(2\right)=0,{\bar{L}}_{d,j},Y_{d}=0\right)\times P\left(A_{d,j}\left(2\right)=0|\right.\\ & \;\left.\times {\bar{A}}_{d,j-1}\left(1\right),{\bar{A}}_{d,j-1}\left(2\right)=0,{\bar{L}}_{d,j},Y_{d}=0\right), \end{array}$
where ${\bar{A}}_{d,j-1}\left(1\right)\equiv \emptyset$ if *j* − 1 < *d*. This can be done, for example, using a logistic regression to model exposure at each time point as a function of previous exposure and covariates within the *d*th subset. Similarly, a logistic regression can be used to model the censoring variable.
**Step 2:** Construct the unstabilized or stabilized inverse of the treatment mechanism by defining *λ*_*d*_ as in Section 3. If stabilized weights are of interest, set $\lambda_{d}=\prod_{j=d}^{d+s-1}P\left(A_{d,j}\left(1\right)|{\bar{A}}_{d,j-1}\left(1\right),A_{d,j}\left(2\right)=0,Y_{d}=0\right)P\left(A_{d,j}\left(2\right)=0|{\bar{A}}_{d,j-1}\left(1\right),{\bar{A}}_{d,j-1}\left(2\right)=0,Y_{d}=0\right)$ . For unstabilized weights, *λ*_*d*_ = 1.
**Step 3:** Summarize treatment trajectories into *J* distinct groups, then assign each individual in one of these groups based on their highest “posterior” probability.
**Step 4:** Specify a weighted generalized estimating equation model using the IPTW as weights. For example, a pooled weighted Poisson regression model of the outcome with the trajectory groups *G* (which were obtained from the LCGA) and time *d* as regressors could be fitted using the augmented dataset. For inferences, a robust variance estimator that accounts for the fact that each individual can contribute multiple observations in the augmented dataset needs to be used. Such a robust estimator is produced by GEE routines. For example, the function *geeglm* from the **R** package *geepack* can be used to fit the robust Poisson model.

#### 5.2.2 G-computation estimator

Another approach to estimate the parameters of an LCGA-HRMSM is g-computation. The IPTW estimator sequentially creates pseudo-populations where *A*_*d*,*t*_ is independent of confounders ${\bar{L}}_{d,t}$ at each time *t* of each time interval *d*. Opposingly, g-computation does not remove the association between the treatment and the counfounders. Instead, the counterfactual expectation is estimated under each possible (fixed) treatment regime in the observational data [24]. For example, we would estimate the conditional expectation of the outcome had everyone in the population been perfectly adherent to statins, that is under ${\bar{A}}_{d}=1,\ldots ,1$ for all *d*. Various algorithms for implementing g-computation have been proposed (e.g., noniterative conditional expectation, iterated conditional expectations) [39, 40]. Here we consider an algorithm based on iterated conditional expectations that has the advantage of not requiring to model the covariates’ distribution. This algorithm for implementing the g-computation estimator of the parameters of LCGA-HRMSMs requires fitting a sequence of outcome models to estimate each counterfactual expectation for all time intervals. Then, each deterministic treatment regime is assigned to a trajectory group using the same LCGA that was fitted on the observed data. To estimate the parameters of LCGA-HRMSMs, the counterfactual expectations are regressed on the trajectory groups and the index of time intervals according to the structural working model specification. In the current application, we are using a weighted log-binomial and a Poisson model with weights *λ*_*n*_. The latter model gives the parameter estimates of the LCGA-HRMSM.

**Table:** 

Fitting Procedure: LCGA-HRMSM with G-computation
**- a. Estimation of counterfactual expectations**
Under each of the 2^*s*^ possible treatment regimes ${\bar{a}}_{d},d=1\ldots K-s+1$ , the g-computation can be implemented as follows:
**Step 0:** Set the initial value of the conditional outcome $Q_{d,d+s}^{{\bar{a}}_{d}}=Y_{d+s}$
Moving backward, for *j* = *d* + *s* − 1, …, *d*:
**Step 1:** With the observed data fit a logistic regression:
$E\left(Q_{d,j+1}^{{\bar{a}}_{d}}|{\bar{A}}_{d,j}\left(1\right),{\bar{A}}_{d,j}\left(2\right)=0,{\bar{L}}_{d,j},Y_{d}=0\right)=\text{expit}\left(\gamma_{0}+\gamma_{1}{\bar{A}}_{d,j}+\gamma_{2}{\bar{L}}_{d,j}\right);$
**Step 2:** Compute the predicted value under the treatment regime up to time *j*:
${\hat{Q}}_{d,j}^{{\bar{a}}_{d}}=\hat{E}\left(Q_{d,j+1}^{{\bar{a}}_{d}}|{\bar{A}}_{d,j}\left(1\right)={\bar{a}}_{d,j}\left(1\right),{\bar{A}}_{d,j}\left(2\right)=0,{\bar{L}}_{d,j},Y_{d}=0\right);$
At the end of the algorithm, compute ${\hat{Q}}_{d,1}^{{\bar{a}}_{d}}=\frac{1}{n_{d}}\sum_{i=1}^{n_{d}}Q_{1,n_{d}}^{{\bar{a}}_{d}}\left(L_{1,i}\right)$ . The quantity ${\hat{Q}}_{d,1}^{{\bar{a}}_{d}}$ is an estimate for the counterfactual outcome under treatment regime ${\bar{a}}_{d}$ , $E\left[Y_{d+s}^{{\bar{a}}_{d}}|Y_{d}=0\right]$ . Stack the 2^*s*^ estimates in a vector *Q*. Note that this fitting procedure makes it easy to integrate parallel computation and fit simultaneously all *K* − *s* + 1 time intervals.
**- b. Prediction of trajectory groups**
Compute the probability of observing each possible treatment regime ${\bar{a}}_{d}\left(1\right)$ under each of the trajectory groups produced by the LCGA model, that is $P\left({\bar{a}}_{d}\left(1\right)|z=j\right)$ . Then, assign each possible treatment regime to the trajectory group that yields the largest conditional (“posterior”) probability.
**- c. Estimation of the parameters of a LCGA-HRMSM and inferences**
To estimate the parameters of the LCGA-HRMSM, the vector of the estimated counterfactual means *Q* is regressed on the predicted trajectory groups using a log-binomial regression or a Poisson regression as a working model with weight *λ*_*n*_.
The standard errors are estimated using block bootstrapping. For each replication of the bootstrap, *n* individuals and their repeated measures across all subsets are selected with replacement. G-computation is then performed on each subset before performing a pooled regression of the counterfactual means onto the trajectory groups. Standard errors are estimated by taking the standard deviation of the vector of the HRMSM parameter estimates over the *B* bootstrap replications. Note that the recourse to bootstrap for estimating standard errors makes the g-computation estimator very computationally intensive.

#### 5.2.3 Pooled LTMLE estimator

The IPTW requires fitting a series of models for the treatment, whereas g-computation requires fitting a series of outcome models. Both estimators are consistent only if all the models involved are correctly specified. Doubly robust approaches, such as pooled LTMLE, combine the strength of both methods to yield consistent estimates if either all the models for the treatment or all the models for the outcome are correct. Moreover, the pooled LTMLE is a locally efficient estimator; in other words, the LTMLE achieves the semiparametric asymptotic bounds which guarantees a minimum asymptotic variance among all regular asymptotically linear (RAL) estimators making the same assumptions on the model space when all models are correctly parametrically specified [41, 42]. In practice, the pooled LTMLE is a flexible algorithm that can easily be combined with machine learning techniques.

The pooled LTMLE defines a plug-in estimator for a target parameter denoted *ψ*. In our context, we denote the LTMLE $\psi^{{\bar{a}}_{d}}$ for the *d*th time interval. The target parameter is expressed as an average over time periods of the time-specific vector of conditional means. The fitting procedure is similar to the fitting procedure for g-computation. As in g-computation, the procedure first entails estimating the 2^*s*^ counterfactual expectations under each possible treatment regime ${\bar{a}}_{d}$ for each of the *K* − *s* + 1 subsets. Each possible treatment regime is then assigned to a trajectory group. In fact, the g-computation estimate serves as an initial estimate in the LTMLE procedure; LTMLE sequentially updates each initial estimate using information from the treatment models that are used in constructing the IPTW estimator. Finally, the estimated counterfactual means are regressed on the predicted trajectory groups and the time interval index using either a log-binomial or a Poisson model, with weight *λ*_*n*_. It is noteworthy that the target parameter *ψ* represents the updated counterfactual means upon which we aim to regress the trajectory groups, in order to estimate our causal parameter of interest *β*.

**Table:** 

LCGA-HRMSM with Pooled LTMLE
**- a. Estimation of updated counterfactual means**
LTMLE estimates are obtained following two major steps: (i) initial estimation of the counterfactual means using a plug-in estimator (i.e., g-computation), (ii) and fluctuation of the initial plug-in estimator with an error such that it solves the equations of the efficient influence curve (EIC) (updating phase) [43, 44].
For each time *j* = *d* + *s* − 1, …, *d*:
**Step 1:** Estimate the product $\prod_{t=d}^{j}g_{d}\left(A_{d,t}\left(1\right)\right)g_{d}\left(A_{d,t}\left(2\right)\right)=\prod_{t=d}^{j}g_{d}\left(A_{t}\left(1\right)|{\bar{A}}_{d,t-1}\left(1\right),A_{d,t}\left(2\right)=0,{\bar{L}}_{d,t},Y_{d}=0\right)g_{d}\left({\bar{A}}_{d,t-1}\left(2\right)=0|{\bar{L}}_{d,t},Y_{d}=0\right)$ .
**Step 2:** For all the 2^*s*^ treatment regimes, set the initial value of the conditional outcome $Q_{d,j+1}^{{\bar{a}}_{d}}=Y_{s+d}$ .
**Step 3:** With the observed data fit a logistic regression for the conditional outcome:
$$E\left(Q_{d,j+1}^{{\bar{a}}_{d}}|Y_{d}=1,{\bar{A}}_{d,j}\left(1\right),{\bar{A}}_{d,j}\left(2\right)=0,{\bar{L}}_{d,j}\right))=\text{expit}\left(\gamma_{0}+\gamma_{1}{\bar{A}}_{d,j}+\gamma_{2}{\bar{L}}_{d,j}\right);$$
**Step 4:** For all 2^*s*^ treatment regimes, compute the predicted value:
$${\hat{Q}}_{d,j}^{{\bar{a}}_{d}}=\hat{E}\left(Q_{d,j+1}^{{\bar{a}}_{d}}|{\bar{A}}_{d,j}\left(1\right)={\bar{a}}_{d,j},{\bar{A}}_{d,j}\left(2\right)=0,{\bar{L}}_{d,j}={\bar{l}}_{d,j},Y_{d}=0\right);$$
**Step 5:** For all 2^*s*^ treatment regimes compute the clever covariates:
$$H_{d,j}=\frac{I\left({\bar{A}}_{d,j}\left(1\right)={\bar{a}}_{d,j}\left(1\right),I\left({\bar{A}}_{d,j}\left(2\right)=0\right)\right)}{\overset{j}{\underset{t=d}{\prod }}g_{d}\left(A_{d,t}\left(1\right)\right)g_{d}\left(A_{d,t}\left(2\right)\right)}\lambda \left({\bar{a}}_{d,t}\right)\frac{\partial }{\partial \beta }m\left({\bar{a}}_{d}|\beta \right){\mathrm{Var}}^{-1}m\left({\bar{a}}_{d}|\beta \right).$$
The term $\frac{\partial }{\partial \beta }m\left({\bar{a}}_{d}|\beta \right){\mathrm{Var}}^{-1}m\left({\bar{a}}_{d}|\beta \right)$ corresponds to the vector (1, *z*_1_, *z*_2_ … , *z*_*J*−1_) for the derivative taken with respect to *β*_0_, *β*_1_… and *β*_*J*−1_.
Therefore, the dimension of the weights **H**_*d*_ for all *j* in the pooled TMLE is *n* × 2^*s*^ × dim(*β*).
**Step 6:** Estimate the vector of fluctuation errors *ϵ* by fitting a pooled logistic regression over all 2^*s*^ treatment regimes on **H**_*d*_ without an intercept and with ${\hat{Q}}_{d,j+1}^{{\bar{a}}_{d}}$ as an offset.
$$\text{logit}\left(Q_{d,j}^{{\bar{a}}_{d}^{*}}\right)=\text{logit}\left({\hat{Q}}_{d,j+1}^{{\bar{a}}_{d}}\right)+\epsilon_{d}H_{d}.$$
The final step of the algorithm consists of updating the target parameter with:
$$Q_{d,j}^{{\bar{a}}_{d}^{*}}=\text{expit}\left(\text{logit}\left({\hat{Q}}_{d,j+1}^{{\bar{a}}_{d}}\right)+\epsilon_{d}H_{d}\right).$$
Steps - **b. Prediction of trajectory groups** and - **c. Estimation of the parameters** *β* **of a LCGA-HRMSM**, are performed in the same way as in the g-computation algorithm presented in Section 5.2.2.

##### 5.2.3.1 Variance estimation

Two possible ways to estimate the variance of the pooled LTMLE estimator are to use block bootstrapping as in g-computation or to use the empirical influence curve of the estimator of *β*. However, in the case of an HRMSMs, using influence curves is not straightforward. Indeed, when using LTMLE to estimate the parameters of a usual MSM (i.e., not an HRMSM), the variance of the estimated target parameter $\hat{\psi }\left(Q^{{\bar{a}}_{t}}\right)\equiv \hat{\psi }$ is given by:

$$\mathrm{Var}\left(\hat{\psi }\right)\approx \mathrm{Var}\left(\frac{1}{n}\overset{n}{\underset{i=1}{\sum }}IF_{i,\beta }\right)=\frac{1}{n}\mathrm{Var}\left(IF_{i,\beta }\right).$$

The EIC is defined as:

$$\begin{array}{ll} IF_{\beta } & =\underset{d\in T_{s}}{\sum }C{\left(Q^{d}\right)}^{-1}\underset{t,{\bar{a}}_{d}}{\sum }H\left({\bar{a}}_{d},t\right)\left({\bar{Q}}^{{\bar{a}}_{d},t}-m\left({\bar{a}}_{d}|\beta \right)\right)+\underset{d\in T_{s}}{\sum }C{\left(Q^{d}\right)}^{-1}\underset{t}{\sum }\underset{k\in I_{d}}{\sum }\underset{{\bar{a}}_{d}}{\sum }H\left({\bar{a}}_{d},t\right)\frac{I\left({\bar{A}}_{d}={\bar{a}}_{d}\right)}{g_{d}}\left({\bar{Q}}^{{\bar{a}}_{d},k+1}-{\bar{Q}}^{{\bar{a}}_{d},k}\right). \end{array}$$

where $C\left(Q^{d}\right)=E_{Q^{d}\left(L_{d,1}\right)}\sum_{t,{\bar{a}}_{d}}H\left({\bar{a}}_{d},t\right)\frac{\partial }{\partial \beta }m\left({\bar{a}}_{d}|\beta \right)$ and $H\left({\bar{a}}_{d},t\right)$ designates the clever covariate function of treatment regime ${\bar{a}}_{d}$ at time *t*. The target parameter $\psi^{{\bar{a}}_{d}}$ obtained with the pooled LTMLE solves the EIC equation $P_{n}IF_{\beta }\left({\bar{Q}}_{n}^{{\bar{a}}_{d}^{*}},g_{n},\psi^{{\bar{a}}_{d}}\left(Q_{n}^{{\bar{a}}_{d}^{*}},Q_{L_{d,1},n}^{{\bar{a}}_{d}^{*}}\right)\right)=0$ [43].

This result holds when the observations are independent and identically distributed. In the case of an HRMSM, a given individual can contribute data to multiple observations (subsets), thus inducing correlation between observations. When estimating the variance using influence functions, we have to account for this correlation. In our setting, because only subjects who are event-free at the start of a given subset are included in that subset, the number of individuals included decreases (*n*_1_ ≥ *n*_2_ ≥ … ≥ *n*_*K*−*s*+1_). Based on Schnitzer et al. [45], we can show that the variance of the estimated target parameter $\hat{\psi }\left(\sum_{d\in T_{s}}Q_{d,1}^{{\bar{a}}_{d}}\right)\equiv \hat{\psi }$ using the influence functions is given by:

$$\mathrm{Var}\left(\hat{\psi }\right)\approx \mathrm{Var}\left(\frac{1}{n}\underset{d\in T_{s}}{\sum }\overset{n_{d}}{\underset{i=1}{\sum }}IF_{id,\hat{\beta }}\right)$$

$$\begin{array}{ll} \mathrm{Var}\left(\hat{\psi }\right) & =\mathrm{Var}\left(\frac{1}{n}\underset{d\in T_{s}}{\sum }\overset{n_{d}}{\underset{i=1}{\sum }}IF_{id}\right)\\ & =\left(1/n^{2}\right)\left[\underset{d\in T_{s}}{\sum }n_{d}\mathrm{Var}\left(IF_{id}\right)+2\underset{d\in T_{s}}{\sum }\underset{d^{\prime }>d}{\sum }n_{d^{\prime }}cov\left(IF_{id},IF_{id^{\prime }}\right)\right]\\ & =\left(1/n^{2}\right)\left[\underset{d\in T_{s}}{\sum }n_{d}\sigma_{d}^{2}+2\underset{d\in T_{s}}{\sum }\underset{d^{\prime }>d}{\sum }n_{d^{\prime }}\rho_{d,d^{\prime }}\right] \end{array}$$

with $\sigma_{d}^{2}=\mathrm{Var}\left(IF_{id}\right)$ and $\rho_{d,d^{\prime }}=cov\left(IF_{id},IF_{id^{\prime }}\right)$ . This derivation assumes that all individuals are independent and identically distributed with variance $\sigma_{d}^{2}$ in each of the *d* = 1, …, *K* − *s* + 1 subsets and *ρ*_*d*,*d*′_ designates the correlation between the influence functions from subsets *d* and *d*′ among observations arising from the same individual. In other words, this derivation allows for correlations between observations from the same individual across subsets, but not for correlations between observations from different individuals, whether within a given subset or between subsets. Note that $\mathrm{Var}\left(IF_{id}\right)=E\left(IF_{id}^{2}\right)$ since *E*(*IF*_*id*_) = 0 by construction. Finally, it is worth noting that the variance estimator based on the EIC is only consistent if both the treatment and outcome models are correctly specified [46].

As a concrete example, with two subsets we get $\sigma^{2}=1/n^{2}\left[n_{1}\sigma_{1}^{2}+n_{2}\sigma_{2}^{2}+2n_{2}\rho_{1,2}\right]$ with $\sigma_{1}^{2}=\mathrm{Var}\left(IF_{i1}\right)$ , $\sigma_{2}^{2}=\mathrm{Var}\left(IF_{i2}\right)$ and $\rho_{1,2}=cov\left(IF_{i1},IF_{i_{2}}\right)$ .

## 6 Simulation study

### 6.1 Description

We now present a simulation study that aims to investigate the relative performance of four estimators of the parameters of an LCGA-HRMSM: unstabilized IPTW, g-computation, pooled LTMLE and pooled LTMLE combined with SuperLearner denoted pooled LTMLE + SL. As algorithms for the SuperLearner, we used a generalized additive model (GAM) and generalized linear model (GLM). We also present the crude unadjusted model. The data-generating mechanism is adapted from [32]. The data generated are of the form (*V*, *Y*_1_, *L*_1_, *A*_1_, *Y*_2_, *L*_2_, *A*_2_, *Y*_3_, *L*_3_, *A*_3_, *Y*_4_, *L*_4_, *A*_4_, *Y*_5_, *L*_5_, *A*_5_, *Y*_6_) as presented in Section 4. Confounders *V*, *L*_*t*_, *t* = 1, 2, 3, 4, 5 are binary variables and *V* ⊆ *L*_1_ is a set of baseline variables. The outcome variable *Y*_*t*_, *t* = 1, 2, 3, 4, 5, 6 indicates the occurrence (*Y*_*t*_ = 1) or absence (*Y*_*t*_ = 0) of an event at time *t*. The exposure *A*_*t*_, *t* = 1, 2, 3, 4, 5 is binary. In this simulation, the outcome is measured at times *t* = 1, 2, …, 6 but only outcomes measured at times *t* = 4, 5 and 6 are considered as dependent variables (*Y*_1_ = *Y*_2_ = *Y*_3_ = 0).

We considered scenarios with 1, 2 and 3 windows of size *s* = 3. We summarized the observed treatment trajectories using 3 trajectory groups. We generated 1,000 datasets of size 5,000 for the observed data. To determine the true values of the parameters, we simulated a population based on the t-specific counterfactual framework by generating 1,000 datasets of size 5,000 for each of the 2^3^ treatment regimes. In the classical counterfactual framework, counterfactual data would be generated using the same equations as those used to generate the observed data except that treatment regimes would be fixed instead of random. In the t-specific counterfactual framework, the same principle is applied but only treatment regimes between time point *t* − *s* + 1 and *t* are deterministic; treatment regimes between time points 1 and *t* − *s* are left random.

For g-computation and pooled LTMLE/pooled LTMLE + SL (Q models), we estimated the mean counterfactual outcome using a logistic regression (see Appendix for the model specification). For g-computation, the standards errors were estimated with 50 replications of block bootstrapping (a number of replications between 50 and 200 is recommended for the estimation of standard errors; see [47] for reference) and for pooled LTMLE/pooled LTMLE + SL, the standards errors were estimated using the influence functions. We considered as working models the log-binomial and Poisson models. For each estimation method, we evaluated the performance by measuring the bias, the standard errors of the estimates (SEE) and the coverage probability of the 95 % confidence intervals.

### 6.2 Data-generating mechanism

We present here the data-generating mechanism adapted from [32].

*Y*_1_ = 0

V∼N(0,1)/4+1

*A*_1_ ∼ *Bernoulli*(expit(−0.5 + 2.5*V*))

*L*_1_ ∼ *Bernoulli*(expit(1 + *V* + 0.5*A*_1_))

*Y*_2_ = 0

*A*_2_ ∼ *Bernoulli*(expit(−0.5 + *V* + 1.2*L*1))

*L*_2_ ∼ *Bernoulli*(expit(1 + *L*_1_ + 0.5*A*_2_))

*Y*_3_ = 0

*A*_3_ ∼ *Bernoulli*(expit(−0.5 + *V* + 1.2*L*_2_))

*L*_3_ ∼*Bernoulli*(expit(1 + *L*_2_ + 0.5*A*_3_))

$Y_{4}∼\left\{\begin{array}{c} 1\;\;\text{if}\;Y_{3}=1\;\\ \text{Bernoulli}\left(1-\text{expit}\left(1+V-0.7L_{3}-0.5A_{3}\right)\right)\;\;\text{if}\;Y_{3}=0\; \end{array}\right.$

*A*_4_ ∼ *Bernoulli*(expit(−0.5 + *V* + 1.2*L*_3_)).

*L*_4_ ∼ *Bernoulli*(expit(1 + *L*_3_ + 0.5*A*_4_))

$Y_{5}∼\left\{\begin{array}{c} 1\;\;\text{if}\;Y_{4}=1\;\\ \text{Bernoulli}\left(1-\text{expit}\left(0.8V-0.7L_{4}-0.5A_{4}\right)\right)\;\;\text{if}\;Y_{4}=0\; \end{array}\right.$

*A*_5_ ∼ *Bernoulli*(expit(−0.5 + *V* + 1.2*L*_4_)).

*L*_5_ ∼ *Bernoulli*(expit(1 + *L*_4_ + 0.5*A*_4_))

$Y_{6}∼\left\{\begin{array}{c} 1\;\;\text{if}\;Y_{5}=1\;\\ \text{Bernoulli}\left(1-\text{expit}\left(0.5V-0.7L_{5}-0.5A_{5}\right)\right)\;\;\text{if}\;Y_{5}=0\; \end{array}\right.$

### 6.3 Specification of the models for the outcome and the treatment

For 1 time interval

$$\begin{array}{ll} & g_{1}:A1∼V\\ & g_{2}:A2∼A1+L1+V\\ & g_{3}:A3∼A2+A1+L1+L2+V \end{array}$$

$$\begin{array}{ll} & Q_{1}:Q2∼A1+L1+V\\ & Q_{2}:Q3∼A2+A1+L2+L1+V\\ & Q_{3}:Y∼A3+A2+A1+L3+L2+L1+V \end{array}$$

For 2 time intervals

$$\begin{array}{ll} & g_{1}:A1∼V\\ & g_{2}:A2∼A1+L1+V\\ & g_{3}:A3∼A2+A1+L1+L2+V\\ & g_{4}:A4∼A3+A2+A1+L1+L2+L3+V|Y4=0 \end{array}$$

$$\begin{array}{ll} & Q_{1}:Q2∼A1+L1+V1+V2+V3\\ & Q_{2}:Q3∼A2+A1+L2+L1+V1+V2+V3\\ & Q_{3}:Y∼A3+A2+A1+L3+L2+L1+V1+V2+V3 \end{array}$$

For 3 time intervals

$$\begin{array}{ll} & g_{1}:A1∼V\\ & g_{2}:A2∼A1+L1+V\\ & g_{3}:A3∼A2+A1+L1+L2+V\\ & g_{4}:A4∼A3+A2+A1+L1+L2+L3+V|Y4=0\\ & g_{5}:A5∼A4+A3+A2+A1+L1+L2+L3+L4+V|Y5=0 \end{array}$$

$$\begin{array}{ll} & Q_{1}:Q2∼A1+L1+V1+V2+V3+V4+V5\\ & Q_{2}:Q3∼A2+A1+L2+L1+V1+V2+V3+V4+V5\\ & Q_{3}:Y∼A3+A2+A1+L3+L2+L1+V1+V2+V3+V4+V5 \end{array}$$

### 6.4 Results

Results of the scenarios with 1, 2 and 3 time intervals are shown respectively in Tables 1–3. In the scenario with a single time interval (Table 1), all estimation methods yielded unbiased estimates, except for the crude model. Moreover, results obtained when using a log-binomial or a Poisson loss function were almost identical. In the other scenarios, g-computation and pooled LTMLE/pooled LTMLE + SL also yielded estimates with little to no bias for all coefficients. The IPTW estimates were overall slightly more biased, particularly in the Scenario with 2 time intervals when employing a log-binomial loss function. This may be due to the convergence issues that were encountered in this specific case. This bias decreased in simulations with a larger sample size (see Appendix 1.4) Of note, the bias was similar for both pooled LTMLE implementations, regardless if SuperLearner was used or not.

**Table 1:** Simulation study results with 1 time interval.

	Log-binomial model			Poisson model		
	Bias	SEE	C95 %	Bias	SEE	C95 %
**Group 1**						
IPTW	0.00	0.11	94 %	0.00	0.11	94 %
G-computation + bootstrap	−0.01	0.10	90 %	−0.01	0.10	90 %
Pooled LTMLE	0.00	0.10	98 %	0.00	0.10	98 %
Pooled LTMLE + SL	0.00	0.10	98 %	0.00	0.10	98 %
Crude model	−0.04	0.14	80 %	−0.04	0.14	80 %
**Group 2**						
IPTW	0.00	0.08	94 %	0.00	0.08	94 %
G-computation + bootstrap	−0.01	0.06	92 %	−0.01	0.06	92 %
Pooled LTMLE	−0.01	0.06	100 %	−0.01	0.06	100 %
Pooled LTMLE + SL	0.00	0.06	100 %	0.00	0.06	100 %
Crude model	−0.14	0.05	9 %	−0.14	0.05	9 %

Bias, *SEE* and C95 % indicate respectively the bias, the standard errors of the estimates and the coverage probability of the 95 % confidence interval. We considered three trajectory groups; the group with the lowest adherence is the reference group (group 3).

**Table 2:** Simulation study results with 2 time intervals.

	Log-binomial model			Poisson model		
	Bias	SEE	C95 %	Bias	SEE	C95 %
**Group 1**						
IPTW	−0.07	0.11	100 %	−0.02	0.10	92 %
G-computation + bootstrap	0.00	0.10	95 %	0.00	0.10	95 %
Pooled LTMLE	0.00	0.10	100 %	0.00	0.10	100 %
Pooled LTMLE + SL	0.00	0.11	99 %	0.00	0.11	99 %
Pooled LTMLE + bootstrap	0.00	0.11	97 %	0.00	0.11	96 %
Crude model	−0.06	0.12	77 %	−0.06	0.12	77 %
**Group 2**						
IPTW	0.02	0.14	100 %	−0.02	0.07	94 %
G-computation + bootstrap	−0.01	0.05	96 %	−0.01	0.05	96 %
Pooled LTMLE	−0.01	0.05	99 %	−0.01	0.05	99 %
Pooled LTMLE + SL	−0.01	0.05	100 %	−0.01	0.05	100 %
Pooled LTMLE + bootstrap	−0.01	0.05	96 %	−0.01	0.05	96 %
Crude model	−0.12	0.04	12 %	−0.12	0.04	12 %

Bias, *SEE* and C95 % indicate respectively the bias, the standard errors of the estimates and the coverage probability of the 95 % confidence interval. In the case of two time intervals, convergence problems were noted with the log-binomial model. We considered three trajectory groups; the group with the lowest adherence is the reference group (group 3).

**Table 3:** Simulation study results with 3 time intervals.

	Log-binomial model			Poisson model		
	Bias	SEE	C95 %	Bias	SEE	C95 %
**Group 1**						
IPTW	0.00	0.11	88 %	−0.02	0.09	90 %
G-computation + bootstrap	−0.01	0.09	97 %	−0.01	0.09	97 %
Pooled LTMLE	−0.01	0.09	100 %	−0.01	0.09	100 %
Pooled LTMLE + SL	−0.01	0.09	100 %	−0.01	0.09	99 %
Pooled LTMLE + bootstrap	−0.01	0.09	99 %	−0.01	0.09	99 %
Crude model	−0.05	0.11	75 %	−0.05	0.11	75 %
**Group 2**						
IPTW	0.00	0.10	96 %	−0.03	0.07	90 %
G-computation + bootstrap	−0.01	0.05	98 %	−0.01	0.05	98 %
Pooled LTMLE	−0.01	0.05	100 %	−0.01	0.05	100 %
Pooled LTMLE + SL	−0.01	0.05	100 %	−0.01	0.05	100 %
Pooled LTMLE + bootstrap	0.00	0.05	99 %	−0.01	0.05	99 %
Crude model	−0.13	0.04	4 %	−0.13	0.04	4 %

Bias, *SEE* and C95 % indicate respectively the bias, the standard errors of the estimates and the coverage probability of the 95 % confidence interval. We considered three trajectory groups; the group with the lowest adherence is the reference group (group 3).

In all scenarios, the SEE of the different estimators were similar, except for IPTW that had overall greater SEE. Overall, the estimators had a lower SEE in the scenarios with more time intervals (see Table 3). In all scenarios, pooled LTMLE/pooled LTMLE + SL yielded comparable confidence interval coverages, between 94 % and 100 %. IPTW had coverage probabilities between 90 % and 100 % in all scenarios and g-computation yielded coverage probabilities between 89 % and 92 % in the scenario with one time interval (Table 1), and between 95 % and 98 % with the scenarios with two and three time intervals (Tables 2 and 3). The Monte Carlo standard error for the bias estimated as $SEE/\sqrt{R}$ was between 0.001 and 0.004; the Monte Carlo error for the coverage probability estimated as $\sqrt{coverage\times \left(1-coverage\right)/R}$ is between 0 and 0.009 with *R* = 1,000 replications [48].

## 7 Application

**Figure 5:**
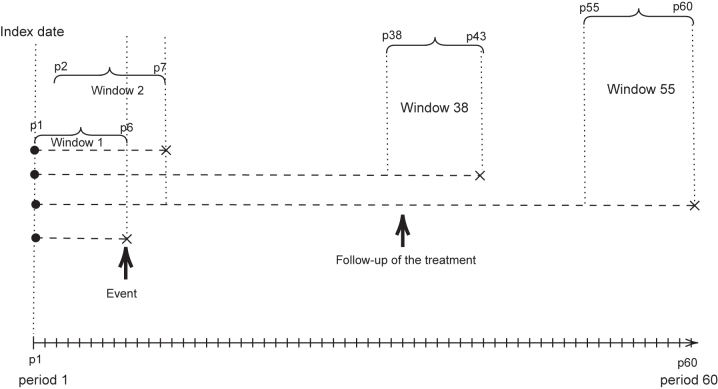
Illustration of the data structure for a follow-up of 60 months (from April 2013 to March 2018) and *p* indicates the follow-up period. Each participant is followed from statin initiation (index date) until occurrence of an event.

We applied our LCGA-HRMSM approach to estimate the effect of statin usage trajectories for primary prevention of CVD or all-cause mortality among older adults using the data described in Section 2. For a better understanding of how the data are organized see Figure 5.

**Figure 6:**
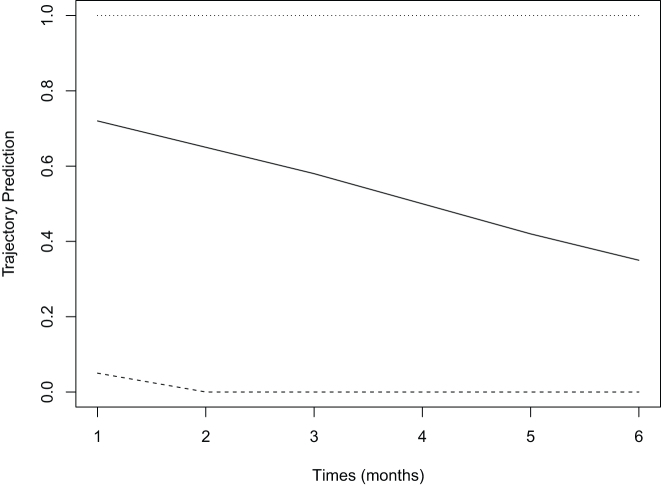
Predicted trajectory groups in the analysis of statin usage for primary prevention among older adults in Quebec, Canada.

The first time interval was composed by 57,211 statin initiators and the last interval by 2,315 individuals who were still alive and CVD-free. A pooled LCGA was fitted with all the individuals at risk at the beginning of each time interval. Treatment trajectories were summarized into 3 trajectory groups and with a log-linear function of time (see Figure 6). We have shown in a previous work with the same dataset that the choice of 3 groups and a linear form was suitable according to the Bayesian Information Criterion (BIC) and the Entropy Information Criterion (see Diop et al. [10]). Groups were ranked from highest to lowest adherence. The group with the lowest adherence was considered as the reference group. To estimate the relative risks, we considered log-binomial and Poisson working models. We also considered all three adjustment methods: IPW (product of IPTW and inverse probability of censoring weights (IPCW)), g-computation and pooled LTMLE. For the bootstrap 100 replications were considered to estimate the standard errors and construct the 95 % confidence intervals.

### 7.1 Results for the marginal models

The mean of adherence across all windows was 0.99 in the first group, 0.54 in the second group and 0.02 in the third group. Overall, the data seemed to indicate a decreasing risk of CVD/all-cause mortality events among participants with a better statin adherence (see Table 4). The estimated relative risk is lower than 1 for the group with the highest adherence compared to the reference group with the lowest adherence for all three estimation methods: IPW, g-computation and pooled LTMLE (IPW – log-binomial: RR = 0.77; Poisson: RR = 0.76; g-computation, log-binomial: RR = 0.65 and Poisson: RR = 0.66; pooled LTMLE, log-binomial and Poisson RR = 0.75). However, the 95 % confidence interval is wider when estimating the relative risk with IPW and includes 1, 95 % CI: [0.53, 1.10] for both log-binomial and Poisson models, suggesting that the effect of a better adherence to statins might also be null or detrimental up to a relative risk of 1.10. For g-formula and pooled LTMLE the 95 % confidence intervals were 95 % CI: [0.57, 0.75] and 95 % CI: [0.59, 0.97] both suggesting beneficial effects of a high statin adherence over a low adherence.

**Table 4:** Application of LCGA-HRMSM to estimate statin usage trajectories for primary prevention of CVD or all-cause mortality among older adults using IPW, g-computation and pooled LTMLE. Both log-binomial and Poisson were considered as working models with group 1 being the group with the lowest adherence probability followed by group 2.

Log-binomial					Poisson			
	RR	SE	Lower 0.95	Upper 0.95	RR	SE	Lower 0.95	Upper 0.95
**Crude model**								
Group 1	0.87	0.10	0.71	1.06	0.87	0.10	0.71	1.19
Group 2	1.57	0.13	1.20	2.06	1.56	0.14	1.06	2.04
**IPW**								
Group 1	0.77	0.19	0.53	1.10	0.76	0.19	0.53	1.10
Group 2	1.07	0.23	0.69	1.67	1.04	0.23	0.67	1.64
**G-computation + bootstrap**								
Group 1	0.65	0.07	0.57	0.75	0.66	0.08	0.58	0.77
Group 2	0.81	0.27	0.48	1.39	0.83	0.27	0.49	1.40
**Pooled LTMLE**								
Group 1	0.75	0.13	0.59	0.97	0.75	0.13	0.59	0.97
Group 2	0.89	0.33	0.47	1.71	0.89	0.33	0.47	1.71

RR, relative risk; SE, standard errors; lower 0.95 and upper 0.95 the lower and the upper bounds of 95 % confidence intervals.

For the group with the second highest adherence to statins, 95 % confidence intervals obtained with IPW, g-formula and pooled LTMLE included 1. More precisely, the relative risk when comparing the second group to the reference group was close to 1 when using the IPW (log-binomial: RR = 1.07, 95 % CI: [0.69, 1.67]; Poisson: RR = 1.04, 95 % CI: [0.67, 1.64]). The estimated relative risk was below 1 when using either g-computation or pooled LTMLE (RR = 0.81, 95 % CI: [0.48, 1.39]; RR = 0.89, 95 % CI: [0.47, 1.71]).

## 8 Discussion

In this study, we proposed an extension of the LCGA-MSM framework to a time-dependent outcome using a nonparametric HRMSM. LCGA is used to summarize treatment trajectories into few trajectory groups; then HRMSM is used to relate the trajectory groups to the time-dependent outcome. HRMSMs are seen as a generalization of a standard MSM and allow modeling the risk of an event at each time interval according to the recent trajectory. As far as we know, we present the first application of HRMSMs with a time-to-event outcome. It was previously noted that HRMSMs could pose interpretation problems in survival analysis when either targeting a hazard ratio or a survival curve [16]. To bypass these interpretation challenges, we proposed as causal parameter the absolute risk. We considered a weighted log-binomial and Poisson working models to estimate the absolute risk with weight *λ*_*n*_. To estimate the parameters of an LCGA-HRMSM, we used unstabilized IPTW, g-computation and pooled LTMLE without and with SuperLearner. We also proposed an approach to estimate the variance based on influence functions when using the pooled LTMLE.

We conducted a simulation study to assess the empirical performance of the proposed LCGA-HRMSM estimators. Overall, results are similar when considering a log-binomial or a Poisson working model. For all scenarios, we obtained unbiased estimates when using either g-computation or pooled LTMLE/pooled LTMLE + SL. This result was particularly expected with the pooled LTMLE as it is a doubly robust method. Unstabilized IPTW had a less good performance when considering two time intervals. Indeed, IPTW is well known in the literature for inducing more variability in the estimation of parameters. All approaches had good coverage of the 95 % confidence intervals. Estimates of the variance after correction for the pooled LTMLE are similar to the estimates of the variance obtained trough block-bootstrapping. This result validates our proposed correction for the dependence between influence functions. We also applied our approach to a population of 57,211 statin initiators to investigate the benefits of using statin for primary prevention of CVD or death events among older Quebecers. We found that the estimates obtained with the g-computation and pooled LTMLE lead to the same conclusions that high adherence to statins might be beneficial for primary prevention and reduce the risk of CVD or death among older adults. However, the IPW gave a slightly different, more inconclusive, result. For an average statin adherence, the three estimation methods produce inconclusive results, since the 95 % confidence intervals show that the data are compatible with both clinically meaningful beneficial and detrimental associations.

Along with the strength of LCGA-HRMSMs, there are some limitations. In practice, a user can encounter challenges regarding the choice of the hyperparameters *s* which is the size of a time interval or *J* the number of trajectory groups. With the wrong choice of *s*, estimation problems might arise. For example, this can happen when in a time interval all individuals are exposed or unexposed. Verification can be done for all time intervals, to verify if the chosen *s* allows a good distribution of the data. As for the choice of the number of groups *J*, it can be chosen based on different criterias as the Bayesian information criterion, through cross-validation or bootstrap [11, 49, 50]. As argued in Nagin [11] and Diop et al. [10], trajectory groups are not meant to represent the true data-generating distribution but can be seen as points of support. Another limitation is that the LCGA step assumes that missingness in the treatment trajectory is MAR conditional on the groups. If this assumption does not hold, the LCGA may fail to identify the true trajectory groups, if trajectory groups truly exist. However, because we take the perspective that the trajectory groups are a convenient approximation to the truth, and define our estimand conditional on the groups that are formed, we do not see this limitation as fundamental. Nonetheless, extensions that relax the assumptions on the missingness mechanism for the LCGA step may be an interesting direction for future work. For example, Vermunt et al. [34] discuss ways of accommodating not missing at random data.

We believe that LCGA-HRMSM is an interesting and useful approach when one needs to investigate the effect of different adherence profiles on a time-dependent outcome. In this paper, our focus was on primary intervention. Thus, we did not take into account recurrent events. However, our approach LCGA-HRMSM is easily extendable to the case where we encounter multiple events which makes it interesting for more complex settings. Moreover, important gains in computation time can be made when using g-computation or pooled LTMLE/pooled LTMLE + SL as the fitting procedure can be applied simultaneously on all time intervals using, for example, parallel computation.

## Supplementary Material

Supplementary Material Details
